# Assessment of left atrial myocardial deformation using two-dimensional speckle-tracking echocardiography in cats with cardiogenic and non-cardiogenic arterial thromboembolism

**DOI:** 10.1080/23144599.2023.2196853

**Published:** 2023-03-31

**Authors:** Jidapa Tosuwan, Sirilak Disatian Surachetpong, Vachira Hunprasit

**Affiliations:** Department of Veterinary Medicine, Faculty of Veterinary Science, Chulalongkorn University, Bangkok, Thailand

**Keywords:** Arterial thromboembolism, feline, left atrium, myocardium, speckle tracking

## Abstract

Arterial thromboembolism (ATE) in cats usually relates to cardiogenic causes that can be diagnosed by conventional echocardiography. Two-dimensional speckle-tracking echocardiography (2D-STE) is a new ultrasound modality with higher sensitivity. Our objective was to investigate left atrial myocardial deformation in cats with both cardiogenic and non-cardiogenic ATE and normal cats using 2D-STE. Twenty-three normal cats and 21 cats with ATE cats were recruited and performed conventional echocardiography and 2D-STE. From the results, left atrial (LA) strain and strain rate from 2D-STE were significantly decreased in cats with cardiogenic ATE (*P* < 0.001), but there was no significant difference in non-cardiogenic ATE compared with normal cats. From the correlation test, the use of left atrial strain during the reservoir phase (LASr) could represent the overall LA deformation. The intra- and inter-observer coefficient of variation of LASr was less than 15%. The logistic regression revealed that the LASr value of less than 11% was a significant factor for the occurrence of ATE (odd ratio = 189.0, *P* < 0.001). In conclusion, LASr derived by 2D-STE is a repeatable and non-invasive technique to assess LA myocardial deformation in cats with ATE. By 2D-STE, impaired LA function was detected in cats with cardiogenic ATE. LASr < 11% may use as a predictor of the risk of ATE occurrence in cats.

## 1. Introduction

Feline arterial thromboembolism (ATE) is an acute and distressing clinical presentation with high mortality and poor prognosis. ATE can be classified as cardiogenic arterial thromboembolism (CATE) and may be related to non-cardiogenic causes such as hyperthyroidism and neoplasms, particularly pulmonary carcinoma [1] [2,3]. A previous study has shown that ATE occurs primarily in cats with hypertrophic cardiomyopathy (HCM) and other cardiomyopathies [4]. Factors associated with CATE include left atrial (LA) enlargement, LA systolic dysfunction, increased left ventricular (LV) wall thickness and diameter, decreased left atrial appendage (LAA) flow velocities and appearance of spontaneous echocardiographic contrast (SEC) in left atrium [4–6]. The pathogenesis of LA thrombus formation is still unclear [6,7]. Left atrial dysfunction could be one of the causes of thrombus formation in LA. Therefore, evaluation of left atrial function may be useful to identify cats at risk for developing ATE. Echocardiography is the primary diagnostic tool for myocardial disease in cats, detecting structural changes and functional abnormalities of the myocardium in a non-invasive manner. Conventional echocardiography (two-dimensional (2D), M-mode, and Doppler echocardiography) is routinely used to diagnose cardiomyopathies in cats [8]. Two-dimensional speckle-tracking echocardiography (2D-STE) echocardiography, an ultrasound modality based on 2D echocardiographic images, allows multidirectional active deformation assessment and providing comprehensive information on myocardial contractile properties and function. 2D-STE echocardiography is used to estimate the strain and strain rate of radial, longitudinal and circumferential myocardial deformation. 2D-STE is recognized as an accurate technique for assessing myocardial function and has been used in many studies in humans [9,10] and dogs [11–13]. In cats with HCM, 2D-STE has been used in many studies to assess left ventricular dysfunction and can assess contractile function in more detail [14–16].

ATE prevention is better than treatment. If the abnormal myocardial function associated with ATE can be identified, this may lead to opportunities for ATE prevention. The use of 2D-STE may facilitate early prediction or diagnosis of atrial dysfunction rather than prevention of ATE. In addition, a previous investigation examined the correlation between recovery outcome and the time of admission for cats with ATE [17]. To our knowledge, evaluation of left atrial function by 2D-STE in cats with ATE has not been reported previously. Therefore, the aim of this study is to evaluate left atrial deformation in cats with ATE and normal cats using 2D-STE.

## 2. Materials and methods

This case–control study was reviewed and approved by the Institutional Animal Care and Use Committee (IACUC) of the Faculty of Veterinary Science, Chulalongkorn University with ethical approval number 2,031,033 on 1 August 2020.

### 2.1. Definitions and data acquisition

Cats with ATE were defined as limb paralysis with pulselessness, pain, poikilothermia, and pallor of the extremities. The confirmation of ATE was obtained by evaluating the arterial flow using a Doppler device [3]. To provide further evidence of ATE, a comparison of glucose and potassium levels was conducted between the affected and non-affected limbs. Whereas clinically normal cats had to have a normal physical examination, blood tests, a normal cardiac structure and function, and no previously diagnosed disease. The population included client-owned cats with ATE and normal cats presented to the Small Animal Teaching Hospital, Faculty of Veterinary Science, Chulalongkorn University, Thailand, during 2020–2021. Adult cats, older than one-year-old [18], bodyweight between 2 and 7 kilograms of any breed and sex were recruited for the study. Data including age, sex, breed, body weight, body condition score, disease history, and clinical findings were collected from all cats. A complete physical examination, systolic blood pressure, thoracic radiography with vertebral heart score (VHS) measurement [19], conventional electrocardiography, and blood collection were performed in all cats. Complete blood count, blood chemistry, electrolytes, and total T4 were measured. Cats with kidney diseases, systemic hypertension and hyperthyroidism were excluded. Cardiac structure and function were examined by echocardiography. Using conventional echocardiography, ATE cats with cardiomyopathy such as HCM, restrictive (RCM), dilated (DCM) and unclassified cardiomyopathy (UCM), were categorized as cardiogenic ATE (CATE), while cats with ATE without cardiomyopathy including or with other cardiac diseases were classified as non-cardiogenic ATE (non-CATE) [20].

Sample size calculation was estimated by using the trial data of left atrial strain in reservoir phase (LASr) derived from 2D-STE, compared between cardiogenic ATE, non-cardiogenic ATE and normal groups. The calculation was performed using a freeware program (GPower 3.1, Heinrich Heine University Düsseldorf, Germany). For the calculation, the mean of LASr of cardiogenic ATE, non-cardiogenic ATE and normal groups were 2.87, 11.41 and 23.63, respectively. The estimated standard deviation (SD) was set to 10.43, the power was set to 0.80, and α (confidence level or type I error rate) was set to 0.05. The result for sample size calculation was 18 cats (i.e. 6 cats with cardiogenic ATE, 6 non-cardiogenic ATE and 6 normal cats). The calculated sample sizes are approximate to previous studies [14,16,21].

### 2.2. Conventional echocardiography

Conventional echocardiography was performed with an ultrasound machine (Mindray, M9, Shenzhen, P.R. China) by an investigator (SS). M-mode echocardiography was performed to measure the wall thickness and chamber size in the right parasternal long-axis four-chamber view. M-mode parameters including the left ventricular internal diameter at the end-diastole and systole (LVIDd and LVIDs), interventricular septum at the end-diastole and systole (IVSd and IVSs), and left ventricular posterior wall thickness at the end-diastole and systole (LVPWd and LVPWs) were measured. The percentage of fractional shortening of the left ventricle (LV FS) was calculated. Ejection fraction (EF) and LV volumes were calculated as end-diastolic volume (EDV) and end-systolic volume (ESV) [22]. The maximum and minimum dimensions of the left atrium were measured by M-mode echocardiography in the right parasternal outflow tract view. The percentage of fractional shortening of the left atrium (LA FS) was calculated using the formula (LADmax-LADmin/LADmax) x100 [23].

In the right parasternal short-axis view at the level of the left atrium during the early diastole, the dimensions of the left atrium and aorta were measured according to the Swedish method [24]. The ratio of the left atrial to aorta dimensions (LA/AO) was calculated. The ejection fraction of the left atrium (LA-EF%) was calculated from the maximum and minimum LA volumes measured in the left apical four-chamber view [25]. Two-dimensional echocardiographic images of the left apical four-chamber view with three cardiac cycles were recorded with at a maximum of 1,000 frames per second (FPS) for 2D-STE offline analysis (Tissue Tracking Quantitative, Mindray, M9, Shenzhen, P.R. China).

Pulsed-wave Doppler echocardiography was performed to measure isovolumic relaxation time (IVRT), transmitral flow velocity, aortic flow velocity, pulmonic flow velocity, and pulmonary vein flow velocities [22,26]. The required indices from transmitral flow velocities were the peak velocity of the early diastolic wave (E wave), the peak velocity of the late diastolic wave (A wave) and the ratio between E wave and A wave (E/A ratio). The fused E and A waves were excluded.

Tissue Doppler imaging was performed with the gate placed at the septal mitral annulus on the left apical 4-chamber view [22]. The peak velocity of the systolic wave (Sa wave), the peak velocity of the early diastolic wave (Ea wave), and the peak velocity of the late diastolic wave (Aa wave) were determined. In addition, the ratio of the peak velocity of the Ea wave to the peak velocity of the Aa wave and the ratio of the peak velocity of the E wave to the peak velocity of the Ea wave were calculated.

### 2.3. Two-dimensional speckle tracking echocardiography (2D-STE)

**Figure 1.**
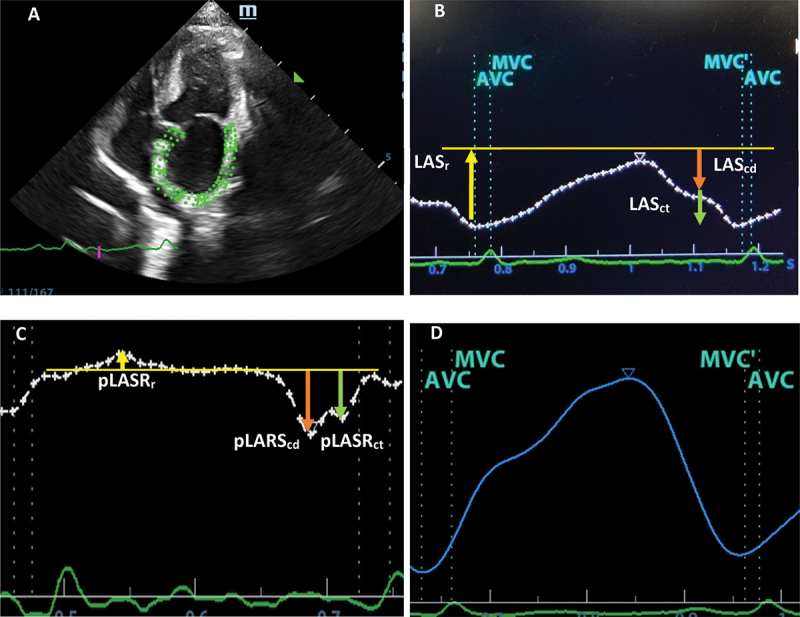
Two-dimensional speckle trackling echocardiography (A) the complete myocardial region of interest of the left atrium (B) Left atrial longitudinal strain curve (C) Left atrial longitudinal strain rate (D) Left atrial volume measurement.

**Figure 2.**
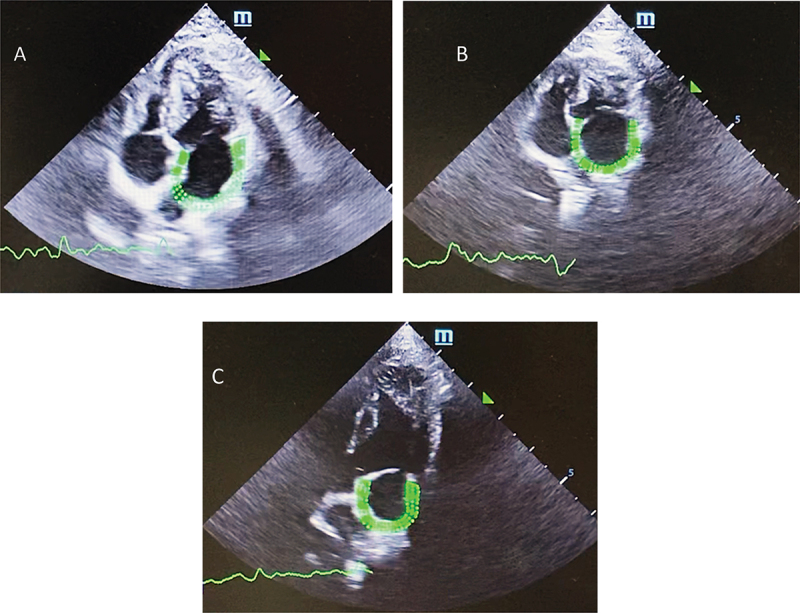
Two-dimensional speckle trackling echocardiography (A) Normal cat (B) Cat with cardiogenic arterial thromboembolism (C) **Cat** with non-cardiogenic arterial thromboembolism.

2D-STE was performed offline in the left apical four-chamber view to analyse the left atrial function by a single operator (JT). Three sets of two-dimensional echocardiographic images stored in DICOM format (Mindray, M9, Shenzhen, P.R. China) were analysed (Figure 1). The complete myocardial region of interest (ROI) of the LA was defined by the endocardial and epicardial borders. Using the left apical four-chamber view, ROI tracing started at the mitral annulus, along the endocardial border, extrapolated across the pulmonary veins and/or the LA appendage orifice, and ended on the opposite side of the mitral annulus using the point-and-click method. An adjustable ROI with a width of 2 mm was used. The size and shape of ROI were then manually adjusted by the operator to account for the LA wall thickness (Figure 2). Strain was analysed from three consecutive cardiac cycles. The strain curve and LA volume including LA EDV, LA ESV and LA EF were automatically computed by the offline software of the ultrasound machine (Figure 1).

Global longitudinal strain was used to assess LA myocardial deformation as left atrial longitudinal strain (LAS) and strain rate (LASR) [27]. LA deformation can be divided into three phases, including reservoir, conduit and contract phases. LAS and LASR were reported separately for each phase by calculating the difference between two measurement points on the strain curve. The left ventricular end-diastole was set as the reference for zero strain by using ECG. LAS result was expressed as a percentage, whereas LASR was expressed as 1/s.

Definitions of LAS in each LA deformation phases [27]

1. LASr = strain during the reservoir phase, measured as the difference in strain values at ventricular end-diastole and at mitral valve opening (positive value).
2. LAScd = strain during conduit phase, measured as the difference in the strain values at mitral valve opening and at the onset of atrial contraction (negative value).
3. LASct = strain during the contraction phase, measured as the difference in strain values at the onset of atrial contraction and at ventricular end-diastole (negative value).

Definitions of the peaks in the LASR curve in each LA deformation phases [27]

1. pLASRr = peak strain rate during the reservoir phase (positive value).
2. pLASRcd = peak strain rate during the conduit phase (negative value).
3. pLASRct = peak strain rate during the contraction phase (negative value).

### 2.4. Statistical analysis

The commercial software IBM SPSS Statistics for Windows, Version 22.0 (IBM Corp., Amonk, NY, USA). Descriptive statistical analysis was performed for cat characteristics including breed and sex in percentage (%). Continuous data were tested for normality using the Kolmogorov–Smirnov test. Initial descriptive statistics included median and interquartile range (IQR) for non-normally distributed data. Differences of general characteristics, conventional echocardiographic and 2D-STE results between the cardiogenic ATE, non-cardiogenic ATE, and normal groups were compared with the Kruskal–Wallis test. Spearman’s rho (r_s_) was used to test the correlation coefficients of the association between LASr and other continuous data of entire samples. The degree of the Spearman’s rho correlation coefficient was determined as follows: r_s_>0.80 means very strong correlation, *r* = 0.60–0.79 means strong correlation; *r* = 0.40–0.59 means moderate correlation; *r* < 0.40 means weak correlation [28].

Logistic regression was performed to develop a predictive model for the occurrence of ATE in the cat population. All categorical factors with a p-value of <0.2 assessed by univariate logistic regression were submitted. Multicollinearity and interaction between parameters were assessed. Parameters with high multicollinearity were excluded. The backward Wald elimination method was used to generate the final model.

Goodness-of-fit statistics were performed to determine whether the model adequately described the data. The model had to meet the majority of the statistical model assumption with the largest Nagelkerke r^2^ [29]. The goodness of the statistical model assumptions for this study was (1) the overall percentage from the classification table >70%, (2) the Hosmer–Lemeshow test was not significant (*P* > 0.05), and (3) the area under the ROC Curve was >0.80. A P-value <0.05 was considered significant. For clinical applicability, we converted LASr to categorical data to obtain the best cut-off value for LASr.

Reliability of measurements was determined using data from six randomly selected cats. The LAS and LASR were used to evaluate the intra- and inter-observer coefficient of variation (CV = standard deviation/mean). The intra-observer coefficient of variation was calculated from three repeated examinations of the same left apical four-chamber video record of each cat on different days (at least 3 days apart) by the same examiner. The inter-observer coefficient of variation was calculated from measurements performed by two different investigators with different levels of experience. The percent CV <15% was accepted in this study [30].

## 3. Results

Forty-four cats participated in this study, including 21 cats with ATE, classified as 15 (76.43%) CATE and 6 (23.57%) non-CATE, and 23 normal cats. The general characteristics of the CATE, non-CATE, and normal cats were analysed descriptively and presented inTables 1 and2. In the CATE group, 10 cats (66.7%) in the CATE had congestive heart failure including 7 cats (46.7%) with pulmonary oedema and 3 cats (20%) with pleural effusion. Concurrent diseases or conditions identified in non-CATE cats in this study included Feline leukaemia virus (FeLV) infection (*n* = 2), Mycoplasma infection (*n* = 1), traumatic scapular fracture (*n* = 1), chronic kidney disease (*n* = 1) and anaemia (*n* = 1). It has not been proven whether these diseases or conditions were the direct cause of ATE in the affected cats. One cat (16.7%) in non-CATE group had pleural effusion. Echocardiographic findings revealed that SEC was present in nine cats (60%) and thrombi within the LA were identified in 3 cats (20%) in the CATE group. While none of the non-CATE cats had SEC and thrombus. Two cats in the CATE group had cardiac arrhythmias, one cat with atrial fibrillation and one cat with ventricular premature complex. Two cats (13.3%) in the CATE group and 5 cats (83.3%) of in the non-CATE group were alive for longer than 7 days. The measurements of conventional echocardiography including M-mode, Doppler and tissue Doppler imaging of CATE, non-CATE, and normal groups are summarized inTable 3.

**Table 1.** Descriptive analysis of general characteristics of cardiogenic arterial thromboembolism (CATE), non-cardiogenic arterial thromboembolism (non-CATE) and normal groups.

Criteria		CATE (*n* = 15)	Non-CATE (*n* = 6)	Normal (*n* = 23)
Sex	Male	12 (80%)	3 (50%)	15 (65.2%)
	Female	3 (20%)	3 (50%)	8 (34.8%)
Breed	DSH	11 (73.3%)	4 (66.8%)	6 (26.09%)
	Persian	1 (6.7%)	1 (16.6%)	3 (13.04%)
	Mixed	2 (13.3%)	-	-
	Maine coon	1 (6.7%)	-	1 (4.35%)
	Scottish	-	1 (16.6%)	10 (43.48%)
	Himalayan	-	-	1 (4.35%)
	Bengal	-	-	1 (4.35%)
	American shorthair	-	-	1 (4.35%)
Heart disease	HCM	12 (80%)	Normal cardiac structure	Normal cardiac structure
	RCM	2 (13.3%)		
	UCM	1 (6.7%)		

Abbreviations: DSH: domestic shorthair; HCM: hypertrophic cardiomyopathy; RCM: restrictive cardiomyopathy; UCM: unclassified cardiomyopathy.

**Table 2.** Medians of general characteristics of cardiogenic arterial thromboembolism (CATE), non-cardiogenic arterial thromboembolism (non-CATE) and normal groups.

Criteria	Cardiogenic ATE (*n* = 15)	Non cardiogenic ATE (*n* = 6)	Normal (*n* = 23)	P-value
Age (years)	5 (4, 8)	3.0 (1.75, 4.0)	4 (1, 6)	0.08
Weight (kg)	4.65 (4.16, 5.09)†	2.5 (3.5, 3.75)	4.2 (3.4, 5.6)	0.031*
VHS	8.45 (7.88, 9,12) §	7.5 (6.8, 8.2)	7.4 (7.0, 7.5)	0.009*
SBP (mmHg)	120 (112.5, 148.75)	100 (96.25, 140.5)	130 (119, 137)	0.404
HR (bpm)	203 (181, 230)	226.5 (209.0, 251.0) ¶	196 (185, 208)	0.025*

Abbreviations: bpm: beats per minute; HR: heart rate; kg: kilograms; SBP: systolic blood pressure; VHS: vertebral heart score.

All measurements were expressed as median (Q1, Q3) and compared with the Kruskal Wallis test.

*The P-value<0.05 represent the significant difference between these 3 groups.

§ indicates the statistically significant difference of pairwise comparison between the CATE groups and the normal group.

¶ indicates the statistically significant difference of pairwise comparison between the non-CATE group and the normal group.

† indicates the significant difference of the pairwise comparison between the CATE group and the non-CATE group.

**Table 3.** Conventional echocardiography results of cardiogenic arterial thromboembolism (CATE), non-cardiogenic arterial thromboembolism (non-CATE) and normal groups.

Criteria	CATE (*n* = 15)	Non-CATE (*n* = 6)	Normal (*n* = 23)	P-value
**2-dimensional measurement**				
LA diameter (cm)	1.52 (1.38, 2.07) ^§, †^	1.18 (1.02, 1.27)	1.12 (1.02, 1.21)	<0.001*
Ao diameter (cm)	0.72 (0.65, 0.79)§	0.72 (0.64, 0.94)	0.85 (0.76, 0.97)	0.019*
LA/Ao	2.29 (1.90, 3.15)§	1.52 (1.30, 1.76)	1.25 (1.15, 1.44)	<0.001*
**M-mode measurement**				
IVSd (cm)	0.53 (0.41, 0.69)	0.45 (0.41, 0.52)	0.45 (0.4, 0.5)	0.162
IVSs (cm)	0.88 (0.63, 0.96)†	0.63 (0.53, 0.66)	0.69 (0.63, 0.77)	0.012*
LVIDd (cm)	1.37 (1.25, 1.59)	1.34 (1.26, 1.37)	1.41 (1.31, 1.46)	0.474
LVIDs (cm)	0.78 (0.43, 0.93)	0.66 (0.57, 0.73)	0.57 (0.44, 0.7)	0.206
LVPWd (cm)	0.62 (0.51, 0.67)§	0.44 (0.36, 0.5)	0.43 (0.39,0.46)	0.001*
LVPWs (cm)	0.78 (0.75, 0.91)†	0.65 (0.61, 0.78)	0.71 (0.63, 0.8)	0.042*
IVS%	43.17 (27.65, 73.78)	37.84 (32.25, 57.48)	53.27 (43.48, 71.87)	0.38
LVPW%	43.33 (24.49, 50.0)§	53.26 (40.01, 79.01)	74.23 (59.72, 84.38)	<0.001*
LV FS (%)	46.25 (39.29, 53.5)§	49.89 (46.14, 56.91)	60.59 (50.83, 66.46)	0.012*
LV EDV (ml)	4.99 (3.74, 7.00)	4.45 (3.87, 4.78)	5.13 (4.23, 5.67)	0.466
LV ESV (ml)	1.06 (0.2, 1.71)	0.67 (0.43, 0.86)	0.45 (0.21, 0.78)	0.218
LV SV (ml)	4.37 (2.97, 5.88)	3.79 (3.26, 4.09)	4.78 (3.73, 5.17)	0.221
LV EF (%)	81.49 (73.56, 88.41)§	84.65 (81.25, 89.96)	91.4 (85.41, 95.07)	0.017*
**Doppler Measurement**				
MV E vel (cm/s)	74.34 (47.57, 94.21)	92.16 (71.66, 105.48)	75.38 (65.33, 86.39)	0.197
MV E PG (mmHg)	2.21 (0.91, 3.58)	3.4 (2.1, 4.5)	2.27 (1.71, 2.99)	0.197
MV A vel (cm/s)	36.41 (30.7, 50.46)§	40.80 (28.76, 64.89)	59.62 (52.31, 68.38)	0.001*
MV A PG (mmHg)	0.58 (0.39, 1.04)§	1.17 (0.55, 1.79)	1.42 (1.09, 1.87)	0.001*
MV E/A	1.67 (1.35, 2.68)§	1.81 (1.41, 2.04)	1.19 (1.1, 1.38)	0.011*
IVRT (s)	0.05 (0.04, 0.06)	0.05 (0.04, 0.05)	0.043 (0.04, 0.049)	0.225
AV vel (cm/s)	81.04 (63.72, 105.48)	77.11 (66.04, 101.66)	90.14 (81.59, 97.67)	0.629
AV PG (mmHg)	2.62 (1.62,4.46)	2.38 (1.79, 4.14)	3.25 (2.67, 3.81)	0.629
PV vel (cm/s)	82.44 (56.94, 95.67)	89.38 (83.89, 103.89)	86.45 (75.69, 92.68)	0.352
PV PG (mmHg)	2.72 (1.29, 3.66)	3.2 (2.82, 4.32)	2.99 (2.29, 3.44)	0.352
Pvein S vel (cm/s)	35.7 (23.25, 42.84)§	34.98 (28.74, 46.19)	53.19 (42.84, 59.97)	0.001*
Pvein D vel (cm/s)	28.7 (23.28, 37.13)§	32.68 (22.31, 39.85)	41.05 (37.84, 47.37)	0.01*
Pvein A vel (cm/s)	14.82 (13.15, 21.69)	12.14 (9.36, 22.51)	16.58 (13.57, 19.99)	0.472
Pvein A dur (s)	0.06 (0.05, 0.08)§	0.05 (0.04, 0.07)	0.045 (0.043, 0.057)	0.03*
Pvein S/D	1.19 (0.87, 1.54)	1.22 (1.00, 1.42)	1.32 (1.17, 1.46)	0.823
**Left atrial measurement**				
LAD (mm)	18.7 (15.05, 22.95) ^§, †^	11.45 (10.63, 14.75)	12.2 (10.9, 14)	<0.001*
LAS (mm)	16.7 (13.4, 21.3)^§, †^	9.00 (8.10, 9.90)	8.5 (7.7, 9.5)	<0.001*
LA FS (%)	9.73 (8.24, 13.89)§	21.79 (20.20, 23.23)	27.17 (25.23, 34.23)	<0.001*
LAA FLOW (cm/s)	18.26 (13.75, 21.25) ^§, †^	52.86 (42.16, 56.25)	35.88 (31.18, 43.13)	0.001*
LAA PG (mmHg)	0.135 (0.082, 0.188)^§, †^	1.12 (0.71, 1.26)	0.53 (0.39, 0.75)	0.001*
**Tissue Doppler Imaging**				
Sa (cm/s)	5.46 (5.07, 6.86)§	7.75 (6.96, 8.55)	8.92 (7.75, 10.33)	<0.001*
Ea (cm/s)	6.96 (6.26, 8.45)§	10.63 (8.0, 12.57)	11.78 (10.76, 13.81)	<0.001*
Aa (cm/s)	5.51 (4.65, 7.6)^§, †^	10.33 (6.63, 13.37)	7.95 (6.56, 9.04)	0.005*
MV Ea/Aa	1.36 (0.69, 1.63)	1.09 (0.70, 1.31)**¶**	1.47 (1.35, 1.78)	0.043*
MV E/Ea	9.01 (6.63, 17.04)§	8.95 (8.26, 11.09)**¶**	6.05 (5.07, 7.31)	<0.001*

Abbreviations: Aa: myocardial velocity associated with atrial contraction; Ao: aorta; AV: atrial valve; dur: duration; E/A: mitral inflow peak E-to-A wave velocities ratio; Ea: early diastolic myocardial relaxation velocity; EDV: LV end-diastolic volume; EF: ejection fraction; ESV: LV end-systolic volume; FS: fractional shortening; HR: heart rate; IVRT: isovolumic relaxation time; IVSd: interventricular septum in diastole; IVSs: interventricular septum in systole; IVS%: percentage of fractional thickening of interventricular septum; LA: left atrial; LA FS: left atrial fractional shortening; LAA FLOW: left atrial appendage flow velocity; LAA PG: left atrial appendage maximal pressure gradient; LAD: left atrial diameter in LA diastole; LAS: left atrial diameter in LA systole; LA/Ao: left atrium to aorta ratio; LV: left ventricle; LVIDd: left ventricular internal diameter in diastole; LVIDs: left ventricular internal diameter in systole; LVPWd: left ventricular posterior wall in end-diastole; LVPWs: left ventricular posterior wall in systole; LVPW%: percentage of fractional thickening of left ventricular posterior wall; SV: stroke volume; MV A: mitral valve peak A wave, MV E: mitral valve peak E wave; MV Ea/Aa: mitral inflow Ea wave-to-Aa wave of tissue Doppler; MV E/Ea: mitral inflow E wave-to-tissue Doppler Ea wave; PG: maximal pressure gradient; PV: pulmonic valve; Pvein A: pulmonary venous A wave; Pvein D: pulmonary venous diastolic wave; Pvein S: pulmonary venous systolic wave; Pvein S/D: pulmonary venous peak systolic-to-diastolic ratio; Sa: systolic myocardial velocity; vel: peak velocity; Vmax: maximal velocity.

All measurements were expressed as median (Q1, Q3) and compared with the Kruskal Wallis test.

*The p-value<0.05 represents the significant difference between these 3 groups.

§ indicates the statistically significant difference of pairwise comparison between the CATE groups and the normal group.

¶ indicates the statistically significant difference of pairwise comparison between the non-CATE group and the normal group.

† indicates the significant difference of the pairwise comparison between the CATE group and the non-CATE group.

Left atrial strain, strain rate, and volume values derived from two-dimensional speckle-tracking echocardiography in the CATE, non-CATE, and normal groups are shown inTable 4. LASr and pLASRr of the CATE group were significantly less positive than the normal group. In addition, LAScd and pLASRcd were significantly less negative than those in the normal group. While LASct and pLASRct in the CATE group were significantly less negative than those in the CATE group compared with the non-CATE and normal groups. LA EDV and LA ESV of the CATE group were also significantly greater than those of the normal group. In addition, LA EF in the CATE group was significantly lower than that in the normal group.

**Table 4.** Left atrial strain, strain rate and volume derived from two-dimensional speckle tracking echocardiography in cardiogenic arterial thromboembolism (CATE), non-cardiogenic arterial thromboembolism (non-CATE) and normal groups.

Criteria	CATE (*n* = 15)	Non-CATE (*n* = 6)	Normal (*n* = 23)	P-value
LASr %	3.30 (2.23, 4.86) §	16.10 (3.95, 25.09)	20.72 (18.68, 25.06)	<0.001*
LAScd %	−1.57 (−3.63, −1.32) §	−6.19 (−9.08, −1.46)	−12.92 (−15.08, −10.73)	<0.001*
LASct %	−1.49 (−2.99, −.89) ^§, †^	−7.86 (−14.43, −2.54)	−8.62 (−12.92, −6.30)	<0.001*
pLASRr (1/s)	0.6 (0.48, 1.21) §	1.78 (0.63, 2.53)	2.04 (1.76, 2.64)	<0.001*
pLASRcd (1/s)	−0.49 (−0.97, −0.30) §	−1.85 (−10.57, −0.73)	−4.25 (−6.26, −3.35)	<0.001*
pLASRct (1/s)	−0.19 (−0.61, −0.08) ^§, †^	−2.49 (−5.60, −0.65)	−2.48 (−3.07, −1.66)	<0.001*
GS %	0.89 (−1.02, 2.71)	15.30 (1.64, 20.97)	10.94 (−5.18, 21.18)	0.082
LA EDV avg (ml)	7.56 (4.29, 13.27) ^§, †^	2.28 (1.55, 3.81)	2.04 (1.28, 2.50)	<0.001*
LA ESV avg (ml)	7.02 (3.7, 12.81) §	1.66 (1.24, 3.30)	1.30 (0.84, 1.47)	<0.001*
LA EF avg %	7.6 (4.0, 9.91) §	19.16 (12.40, 34.72)	35.99 (32.99, 41.04)	<0.001*
TPSD (s)	12.74, (3.16, 49.21)	32.29 (9.29, 49.25)	25.99 (8.05, 43.93)	0.751

Abbreviations: avg: average; cd: during LA conduit phase; ct: during LA contraction phase; EDV: end-diastolic volume; EF: ejection fraction; ESV: end-systolic volume; GS%: global strain; TPSD: total peak systolic dispenser; LAS: left atrial global longitudinal strain; pLASR: peak of left atrial strain rate; r: during LA reservoir phase.

All measurements were expressed as median (Q1, Q3) and compared with the Kruskal Wallis test.

*The P-value<0.05 represent the significant difference between these 3 groups.

§ indicates the statistically significant difference of pairwise comparison between CATE and normal group.

† indicates the significant difference of the pairwise comparison between the CATE group and the non-CATE group.

The correlations between LASr and echocardiographic values assessed by conventional echocardiography and 2D-STE in the entire population are shown in theTable 5.

**Table 5.** Correlation between LASr and echocardiographic values assessed by conventional echocardiography and 2D-STE in entire population (*n* = 44).

Parameters	LASr	
	r_s_	P-value
**Signalment**		
Age	−0.310*	0.041
Weight	−0.238	0.124
**Conventional Echocardiography**		
LA diameter	−0.714**¶**	<0.001
AO diameter	0.257	0.119
LA/Ao	−0.682**¶**	<0.001
IVSd	−0.28	0.065
IVSs	−0.25	0.101
LVIDd	−0.082	0.597
LVIDs	−0.304*	0.045
LVPWd	−0.574**¶**	<0.001
LVPWs	−0.353*	0.019
IVS%	0.094	0.554
LVPW%	0.570**¶**	<0.001
FS	0.424**¶**	0.004
HR	−0.187	0.23
LV EDV	−0.114	0.461
LV ESV	−0.305*	0.044
SV	0.018	0.909
EF	0.409**¶**	0.006
MV E vel	0.123	0.432
MV E PG	0.124	0.427
MV A vel	0.423**¶**	0.008
MV A PG	0.531**¶**	0.001
MV E/A	−0.312	0.053
**Conventional Echocardiography**		
IVRT	−0.178	0.253
AV Vmax	0.218	0.182
AV Pgmax	0.218	0.182
PV Vmax	0.346*	0.031
PV Pgmax	0.343*	0.032
Pvein S Vel	0.515**¶**	0.001
Pvein D Vel	0.447**¶**	0.003
Pvein A Vel	0.105	0.517
Pvein A Dur	−0.422**¶**	0.007
Pvein S/D	0.117	0.465
Sa	0.626**¶**	<0.001
Ea	0.536**¶**	<0.001
Aa	0.445**¶**	0.004
MV Ea/Aa	0.07	0.663
MV E/Ea	−0.330*	0.033
LAD	−0.591**¶**	<0.001
LAS	−0.622**¶**	<0.001
LA FS	0.650**¶**	<0.001
LAA FLOW	0.590**¶**	0.001
LAA PG	0.585**¶**	0.001
**Complete blood count and blood chemistry**		
RBC	0.297	0.056
HCT	0.049	0.759
WBC	−0.317*	0.041
PLATELET	0.272	0.081
ALT	−0.501**¶**	0.001
ALP	−0.199	0.201
Creatinine	−0.297	0.056
BUN	−0.239	0.128
Total protein	−0.159	0.326
Albumin	−0.117	0.485
Total T4	0.231	0.204
**2-Dimentional Speckle Tracking**		
LASr	1	.
LAScd	−0.873**¶**	<0.001
LASct	−0.885**¶**	<0.001
pLASRr	0.717**¶**	<0.001
pLASRcd	−0.848**¶**	<0.001
pLASRct	−0.764**¶**	<0.001
GS %	0.359*	0.017
LA EDV avg (ml)	−0.719**¶**	<0.001
LA ESV avg (ml)	−0.727**¶**	<0.001
LA EF avg %	0.737**¶**	<0.001
TPSD	0.044	0.778

Abbreviations (Conventional echocardiography): Aa: myocardial velocity associated with atrial contraction; Ao: aorta; AV: atrial valve; dur: duration; E/A: mitral inflow peak E-to-A wave velocities ratio; Ea: early diastolic myocardial relaxation velocity; EDV: LV end-diastolic volume; EF: ejection fraction; ESV: LV end-systolic volume; FS: fractional shortening; HR: heart rate; IVRT: isovolumic relaxation time; IVSd: interventricular septum in diastole; IVSs: interventricular septum in systole; IVS%: percentage of fractional thickening of interventricular septum; LA: left atrial; LA FS: left atrial fractional shortening; LAA FLOW: left atrial appendage flow velocity; LAA PG: left atrial appendage maximal pressure gradient; LAD: left atrial diameter in LA diastole; LAS: left atrial diameter in LA systole; LA/Ao: left atrium to aorta ratio; LV: left ventricle; LVIDd: left ventricular internal diameter in diastole; LVIDs: left ventricular internal diameter in systole; LVPWd: left ventricular posterior wall in end-diastole; LVPWs: left ventricular posterior wall in systole; LVPW%: percentage of fractional thickening left ventricular posterior wall; SV: stroke volume; MV A: mitral valve peak A wave, MV E: mitral valve peak E wave; MV Ea/Aa: mitral inflow Ea wave-to-Aa wave of tissue Doppler; MV E/Ea: mitral inflow E wave-to-tissue Doppler Ea wave; PG: maximal pressure gradient; PV: pulmonic valve; Pvein A: pulmonary venous A wave; Pvein D: pulmonary venous diastolic wave; Pvein S: pulmonary venous systolic wave; Pvein S/D: pulmonary venous peak systolic-to-diastolic ratio; Sa: systolic myocardial velocity; vel: peak velocity; Vmax: maximal velocity.

Abbreviations (Complete blood count and blood chemistry): RBC: red blood cell; HCT: haematocrit; WBC: white blood cell; ALT: alanine aminotransferase; ALP: alkaline phosphatase; BUN: blood urea nitrogen.

Abbreviations (2-Dimensional Speckle Tracking Echocardiography): avg: average; cd: during LA conduit phase; ct: during LA contraction phase; EDV: end-diastolic volume; EF: ejection fraction; ESV: end-systolic volume; GS%: global strain; TPSD: total peak systolic dispenser; LAS: left atrial global longitudinal strain; pLASR: peak of left atrial strain rate; r: during LA reservoir phase.

The significant correlation was assessed by Spearman’s rho correlation coefficient.

* Correlation is significant at the 0.05 level (2-tailed).

**¶**Correlation is significant at the 0.01 level (2-tailed).

Logistic regression showed that a LASr value of less than 11% was a significant factor for the occurrence of ATE, with a crude odds ratio of 189.0 (95% CI: 15.73–2,269.86, *P* < 0.001). The Hosmer–Lemeshow test showed the goodness of fit of the model to the data (*P* > .05), the overall percentage of the classification table was 93.2%, the area under the ROC curve was 0.932 and Nagelkerke *r*^2^ was 0.77 (Table 6).

**Table 6.** Logistic regression of LASr and arterial thromboembolism occurrence in cats (*n* = 44).

Variables	Crude OR (95% CI)^a^	*P-*value	Overall percentage (%)^b^	AUC	Nagelkerke r^2^
LASr<4%	42 (4.44–397.01)	0.001	84.1	0.782	0.470
LASr<5/6/7/8/9/10%	87.75 (11.09–649.45)	<0.001	90.9	0.899	0.686
LASr<11%	189.0 (15.73–2269.86)	<0.001	93.2	0.932	0.770

Abbreviations: AUC: area under the ROC curve; CI: confident interval; OD: odd ratio; LASr: left atrial global longitudinal strain during LA reservoir phase.

^a^Crude odds ratio derived for Logistic Regression method with Hosmer–Lemeshow test (*P* > 0.05).

^b^Overall percentage from classification table.

The accuracy of using 2D-STE derived LASr as a predictor of the occurrence of ATE in cats is presented in theTable 7. LASr <11% has higher accuracy compared with other cut-off values.

**Table 7.** Accuracy of LASr from 2D-STE as diagnostic test for arterial thromboembolism occurrence in cats (*n* = 44).

	LASr<11%	LASr<10%	LASr<4%
Sensitivity (%)	93.33	86.67	60.00
Specificity (%)	93.10	93.10	96.55
Prevalence (%)	36.36	34.09	22.73
Positive Predictive Value (%)	87.50	86.67	90.00
Negative Predictive Value (%)	96.43	93.10	82.35
Positive likelihood	13.53	12.57	17.40
Negative likelihood	0.07	0.14	0.41
Overall accuracy (%)	93.18	90.91	84.09

Abbreviations: LASr: left atrial global longitudinal strain during LA reservoir phase.

The intra-observer and inter-observer variability in the measurement of LASr, LAScd, LASct, pLASRr, pLASRcd and pLASRct with 2D-STE in six normal cats are summarized inTable 8. The intra-observer CV was acceptable for all parameters. However, only LASr was acceptable for the inter-observer CV result.

**Table 8.** Intra-observer and inter-observer coefficient of variation of strain and strain rate by using two-dimensional speckle tracking echocardiography (*n* = 6).

Parameters	Intra-observer CV (%)	Inter-observer CV (%)
LASr	5.25	13.71
LAScd	4.17	21.62
LASct	10.8	23.18
pLASRr	9.47	17.08
pLASRcd	8.12	29.07
pLASRct	12.82	27.01

Abbreviations: cd: during LA conduit phase; ct: during LA contraction phase; CV: coefficient of variation; LAS: left atrial global longitudinal strain; pLASR: peak of left atrial strain rate; r: during LA reservoir phase.

## 4. Discussion

Forty-four cats participated in this study. Male cats and HCM were found mainly in the CATE group, which is consistent with previous reports that HCM is the most common cardiomyopathy in cats [20], including in Thailand [31]. Male cats are predisposed to HCM [8]. In this study, domestic shorthair (DSH) cats were the majority of cats with ATE. This finding has been noted in previous studies [1,31,32]. The median body weight of the CATE group was significantly higher than that of the non-CATE group. This was to the participating cat breeds such as Mainecoon, a bigger size breed, within the CATE group. The VHS of the CATE group was higher than that of the normal group suggesting the significant haemodynamic change in the CATE group. Age and systolic blood pressure (SBP) were not different between the three groups. Two cats from the CATE group had cardiac arrhythmias, which may affect cardiac function and deformation. However, according to Badano (2018) [27], LA function can be accurately measured in human patients with atrial fibrillation by using 2D-STE when left ventricular end-diastole is used as a reference for zero strain.

Approximately two-thirds of the CATE group in the present study had congestive heart failure. A similar result was found in a previous study [5]. In the non-CATE group, one of cats had pleural effusion due to cranial mediastinal mass, which might be related to FeLV infection. In addition, SEC was found in 60% of cats in the CATE group. A previous study suggested that the presence of SEC was the factor associated with the occurrence of ATE in cats [33]. None of the animals in the non-CATE group had SEC or thrombus in the heart. The source of thromboembolism in the non-CATE group was unidentified in this study. At 7 days after the onset of ATE, the non-CATE group had higher survival rate than the CATE group.

In the comparisons of echocardiographic values between CATE, non-CATE and the normal group, differences were found only between the CATE and normal groups. These differences could be interpreted as impaired LA function in the CATE group compared with the normal group, which could be due to the increased LA pressure and myocardial dysfunction of cardiomyopathy [8,34]. The median LAA flow in the CATE group was 18.26 cm/s. A previous study has shown that LAA flow less than 20.0 cm/s is associated with increased risk of SEC and ATE [6]. Increased LVPWd and decreased LVPW%, LV FS and LV EF are associated with myocardial thickening and impaired systolic function in cats with HCM [35]. Diastolic function can be measured by LA size, mitral inflow and pulmonary vein flow with conventional echocardiography and TDI [34–39]. In the CATE group, MV A vel, MV A PG, Pvein S vel, Pvein D vel measured by spectral Doppler echocardiography, and Sa, Ea, and Aa measured by TDI technique were decreased with LA enlargement indicating an impaired left ventricular diastolic function compared with the normal group.

The increased LA diameter and decreased Aa, LAA flow and LAA PG of the CATE group showed impaired LA function and increased risk of ATE occurrence (5), which were not found in the non-CATE group. In addition, the non-CATE group had normal LA function as determined by conventional echocardiography.

In the CATE group, left atrial strain and strain rate were less positive during the reservoir phase but less negative during the conduction and contraction phases than in the normal group. These results suggest a decrease in LA deformation in cats with CATE. These findings were similar to those of previous studies examining LA dysfunction in cats with cardiomyopathy [23,34,40]. However, no changes in LA deformation were detected in the non-CATE group, suggesting that the non-CATE group has normal LA deformation and that the thrombus formation in the non-CATE group was not related to LA function. A decreased LA deformation in the CATE group derived from 2D-STE was associated with increased LA volume and decreased LA EF, similar to previous studies [41,42].

The correlation analysis of LASr in all cats showed a very strong negative correlation with LAScd, LASct, pLASRcd, and pLASRct, highlighting the relationship in all 3 LA deformation phases. The use of LASr evaluated by 2D-STE could represent the overall LA deformation. In addition, LASr correlated well with conventional echocardiographic parameters to assess LA systolic function and to determine the LA size, suggesting that LASr can be used as an indicator to assess the LA function in cats. These results are consistent with a previous study [371].

Logistic regression showed that a LASr value of less than 11% was a significant factor for the occurrence of ATE, with a crude odds ratio of 189.0. This was the first study in which LASr was used to predict the risk for the occurrence of ATE. The accuracy of using 2D-STE derived LASr as a diagnostic test for the occurrence of ATE in cats was also analysed. The result showed that the highest accuracy was found when using the cut-off value of LASr <11% compared to other cut-off values.

Intra- and inter-observer variability in measurement of LASr, LAScd, LASct, pLASRr, pLASRcd, and pLASRct by 2D-STE were performed in random three normal and three ATE cats. Intra-observer CV was acceptable for all parameters. However, the inter-observer CV was acceptable only for LASr. This could be related to the tracking procedure. The variability of the tracking procedure could be due to the drop pit of the myocardial wall at the pulmonary vein inlets, the nonadjustable height of the strain and strain rate curves analysed by the STE software and the difficulty of myocardial tracking by the STE software during rapid heart rate measurements. However, LASr may be representative of LA function in cats assessed with 2D-STE, similar to previous studies in cats [42], and dogs [43–45].

This study has some limitations. The first is the limitation imposed by the 2D-STE software. The 2D-STE software was developed for humans, and we adapted it for use in cats. Therefore, the curve height was sometimes very short, and it was difficult to clearly distinguish the phases of strain and strain rate. In addition, strain and strain rates measured with each software varied to some extent. Second, high quality images should be obtained for 2D-STE. The high heart rate and breathing pattern of cats may affect the quality of the images.

In conclusion, the left atrial longitudinal strain of reservoir phase derived by 2D-STE is a repeatable and non-invasive method to assess LA myocardial deformation in cats with ATE. By 2D-STE, impaired LA function was detected in cats with cardiogenic ATE but not in cats with non-cardiogenic ATE. LASr < 11% may predict the risk of occurrence of ATE in cats.
